# Pharmaceutical Companies and Their Drugs on Social Media: A Content Analysis of Drug Information on Popular Social Media Sites

**DOI:** 10.2196/jmir.4357

**Published:** 2015-06-01

**Authors:** Jennifer Tyrawski, David C DeAndrea

**Affiliations:** ^1^The Ohio State UniversityColumbus, OHUnited States

**Keywords:** social media, eHealth, direct-to-consumer advertising, eDTCA, pharmaceutical drugs, online pharmaceutical services, illegal online pharmacies, Facebook, Twitter, YouTube

## Abstract

**Background:**

Many concerns have been raised about pharmaceutical companies marketing their drugs directly to consumers on social media. This form of direct-to-consumer advertising (DTCA) can be interactive and, because it is largely unmonitored, the benefits of pharmaceutical treatment could easily be overemphasized compared to the risks. Additionally, nonexpert consumers can share their own drug product testimonials on social media and illegal online pharmacies can market their services on popular social media sites. There is great potential for the public to be exposed to misleading or dangerous information about pharmaceutical drugs on social media.

**Objective:**

Our central aim was to examine how pharmaceutical companies use social media to interact with the general public and market their drugs. We also sought to analyze the nature of information that appears in search results for widely used pharmaceutical drugs in the United States on Facebook, Twitter, and YouTube with a particular emphasis on the presence of illegal pharmacies.

**Methods:**

Content analyses were performed on (1) social media content on the Facebook, Twitter, and YouTube accounts of the top 15 pharmaceutical companies in the world and (2) the content that appears when searching on Facebook, Twitter, and YouTube for the top 20 pharmaceutical drugs purchased in the United States. Notably, for the company-specific analysis, we examined the presence of information similar to various forms of DTCA, the audience reach of company postings, and the quantity and quality of company-consumer interaction. For the drug-specific analysis, we documented the presence of illegal pharmacies, personal testimonials, and drug efficacy claims.

**Results:**

From the company-specific analysis, we found information similar to help-seeking DTCA in 40.7% (301/740) of pharmaceutical companies’ social media posts. Drug product claims were present in only 1.6% (12/740) of posts. Overall, there was a substantial amount of consumers who interacted with pharmaceutical companies through commenting (23.9%, 177/740). For the drug-specific analysis, we found that the majority of search results contained drug product claims (69.4%, 482/695); more claims mentioned only benefits (44.8%, 216/482) relative to only risks (27.2%, 131/482). Additionally, approximately 25% (150/603) of posts on Twitter and YouTube were presented as personal testimonials. A considerable percentage of content on Facebook contained advertisements for illegal online pharmacies (17%, 16/92).

**Conclusions:**

Pharmaceutical companies avoid making drug product claims on their social media accounts but frequently post content that is consistent with FDA definitions for help-seeking DTCA. Thousands of people often view content posted by pharmaceutical companies on social media; users also share company postings making both direct and indirect influence possible. Finally, people are likely to be exposed to drug product claims and information about illegal pharmacies when searching for information about popular pharmaceutical drugs on social media.

## Introduction

### Background

Direct-to-consumer advertising (DTCA) of pharmaceutical products is an increasingly used but widely debated practice [1,2]. Electronic DTCA (eDTCA), in particular, is a rapidly growing marketing strategy [3] that was recently declared a “global health challenge” [4]. In particular, the features and affordances of social media (ie, interactive Web platforms where users can connect, collaborate, and exchange user-generated content) add complexity to pharmaceutical drug marketing. For instance, pharmaceutical companies can quickly and cheaply reach a variety of consumers online with multimodal, interactive, promotional activities, and consumers can produce promotional content as well [3]. Despite growing concerns about harmful effects, there is a lack of academic research on eDTCA [5]. Given that approximately 75% of adults online in the United States use social media frequently [6], it is critical to examine how social media are being used for eDTCA [4,5]. This study seeks to further our understanding of eDTCA by examining how pharmaceutical companies use social media to interact with the general public and market their drugs.

In addition to pharmaceutical companies’ official social media accounts, it is important to document what information consumers are exposed to when searching popular social media sites for drug information. Researchers have noted that other consumers’ reviews and testimonials are often quite persuasive [3-5]. The extent to which nonexperts make drug efficacy claims and share personal testimonials on social media currently has not been well documented despite the potential for such information to highly influence viewers. Public health officials are also greatly concerned that social media sites are being used to promote or host illegal pharmacies that directly harm patients [3,7,8]. The presence of drug efficacy claims and illegal pharmacies on social media sites is important to examine because these media have the potential to convey a degree of credibility to content they host [9]. Put differently, people might trust the claims made by illegal pharmacies or nonexperts more when the claims are hosted on popular social media sites than on strange or unknown websites. To better understand the prevalence of these concerns and how severely the public might be affected by drug information on social media, we analyzed the nature of information resulting from searches for the 20 most highly sold drugs in the United States on Facebook, Twitter, and YouTube.

### Pharmaceutical Drug Marketing Via Social Media

The practice of DTCA is controversial. Proponents suggest DTCA has positive effects, such as generating disease awareness and increasing patient involvement in health decisions, but opponents suggest DTCA promotes misinformation, overemphasizes the benefits of pharmaceutical treatment over the risks, increases inappropriate prescribing, and more [2,10,11]. Due to these concerns, the US Food and Drug Administration (FDA) regulates the content of DTCA, banning all untruthful or misleading advertisements [2]. Additionally, the FDA requires product claim advertisements, a specific type of DTCA that names the drug and the condition(s) it treats, to present a “fair balance” of the benefits and risks of product use. In print advertisements, pharmaceutical companies must provide a brief summary of all risks associated with product use to meet fair-balance requirements. For broadcast advertisements, a statement of the major risks and information on where to locate complete risk information is required. The 2 other types of DTCA, reminder advertisements and help-seeking advertisements, do not indicate which condition(s) a product treats and thus are not subject to fair-balance rules. Reminder advertisements name the drug and often include information on dosage form or price. Help-seeking advertisements describe a health condition and encourage consumers to discuss the condition and potential treatment options with their doctor.

Online promotional activities, or eDTCA, now occupy an increased share of pharmaceutical companies’ marketing budgets and more companies are marketing through social media [2,3]. Public health researchers have documented the negative effects that can occur from frequent and widespread eDTCA [3,4]. However, it remains unclear how pharmaceutical companies are currently using social media to market their drugs. Prior to changes in Facebook’s commenting policy, many companies had specific social media pages for their products [3,12]. Although most product-specific pages have since been discontinued, pharmaceutical companies still maintain official social media accounts. As such, the first step of this study was to assess the extent to which information akin to the 3 forms of DTCA is present on major pharmaceutical companies’ official social media accounts. We also documented the audience reach of eDTCA and whether companies are adhering to the FDA’s fair-balance guidelines on social media.

In addition to eDTCA shared directly by companies, the interactive nature of social media has raised concerns that consumers might provide inaccurate and dangerous information about drugs on the official social media platforms of pharmaceutical companies [5]. People might be more likely to trust information posted by an outside source, particularly if the source claims to have personal experience with the topic at hand [3-5]. Additionally, pharmaceutical companies can potentially delete or alter negative consumer reviews, leaving only the most flattering portrayals behind [4]. Accordingly, we examined whether pharmaceutical companies provide formal policies that regulate what users can post to their official social media accounts (hereafter user postings/contributions are referred to as “user-generated content”) and the frequency and nature of the posted user-generated content. Specifically, we examine whether users posted personal testimonials about health-related issues, the tone of user-generated comments, and the degree to which companies interacted with consumers.

### Information About Pharmaceutical Drugs on Social Media

Although people can get information directly from pharmaceutical companies’ sites, they can also search for information about particular drugs within popular social media sites. In particular, Facebook, Twitter, and YouTube are 3 of the most common social media platforms [5,6,13,14] that provide search capabilities; in a recent survey, 40% of participants had searched for health information on general social media sites such as these before [13]. The pharmaceutical drug information shared on these sites could have a large impact on their users’ treatment decisions. Specifically, personal testimonials and drug efficacy claims, particularly from people unaffiliated with the pharmaceutical company, can be highly influential [3-5]. What information are people exposed to when they search for pharmaceutical drugs on social media? To address this question, we analyzed the nature of information people are exposed to when searching for the 20 most highly sold drugs in the United States on Facebook, Twitter, and YouTube.

Of critical interest to public health researchers is the extent to which illegal pharmacies are allowed to persist online. Illegal pharmacies are sites where consumers can purchase prescription drugs without a prescription and can compromise public safety by providing drugs to people who have not consulted medical officials and/or by providing counterfeit drugs that are ineffective, lead to injury, or cause death [7,8,12,15]. Given these serious implications for public health safety, we assessed the extent to which people are exposed to illegal pharmacies when searching on popular social media sites for commonly purchased pharmaceutical drugs.

In analyzing the results that appear when people search for pharmaceutical drugs on Facebook, Twitter, and YouTube, we more broadly documented the audience reach of the resulting pages and classified who controls the social media accounts (ie, is the site proprietor the pharmaceutical company or a consumer). We also documented the format and tone of the information posted as well as the nature of the associated user-generated comments.

To summarize, we sought to answer the following research questions:

1. To what extent is eDTCA present on pharmaceutical companies’ social media accounts?
2. What is the nature of the user-generated content present on pharmaceutical companies’ social media accounts?
3. To what extent are (1) drug efficacy claims, (2) personal testimonials, and (3) illegal pharmacies present when searching on popular social media sites for pharmaceutical drugs?

## Methods

Two content analyses (company-specific and drug-specific) were conducted. For the company-specific analysis, the social media content of the top 15 pharmaceutical companies in the global and US Fortune 500 rankings were analyzed [16,17]. The drug-specific content analysis examined information on Facebook, Twitter, and YouTube about the top 20 drugs in 2013 based on US spending [18]. Table 1 lists the pharmaceutical companies and drugs examined.

**Table 1:** Pharmaceutical companies and drugs examined.

Companies	Drugs
Johnson & Johnson	Abilify
Novartis	Nexium
Pfizer	Humira
Roche Group	Crestor
Sanofi	Cymbalta
Merck	Advair Diskus
GlaxoSmithKline	Enbrel
Sinopharm	Remicade
AstraZeneca	Copaxone
Eli Lilly & Company	Neulasta
AbbVie Inc	Rituxan
Bristol-Myers Squibb Co	Lantus SoloSTAR/Lantus
Gilead Sciences, Inc	Spiriva Handihaler
Biogen Idec Inc	Atripla
Mylan Inc	Januvia, Avastin, OxyContin, Lyrica, Epogen, and Celebrex

### Sample

#### Company-Specific Analysis

**Figure 1:**
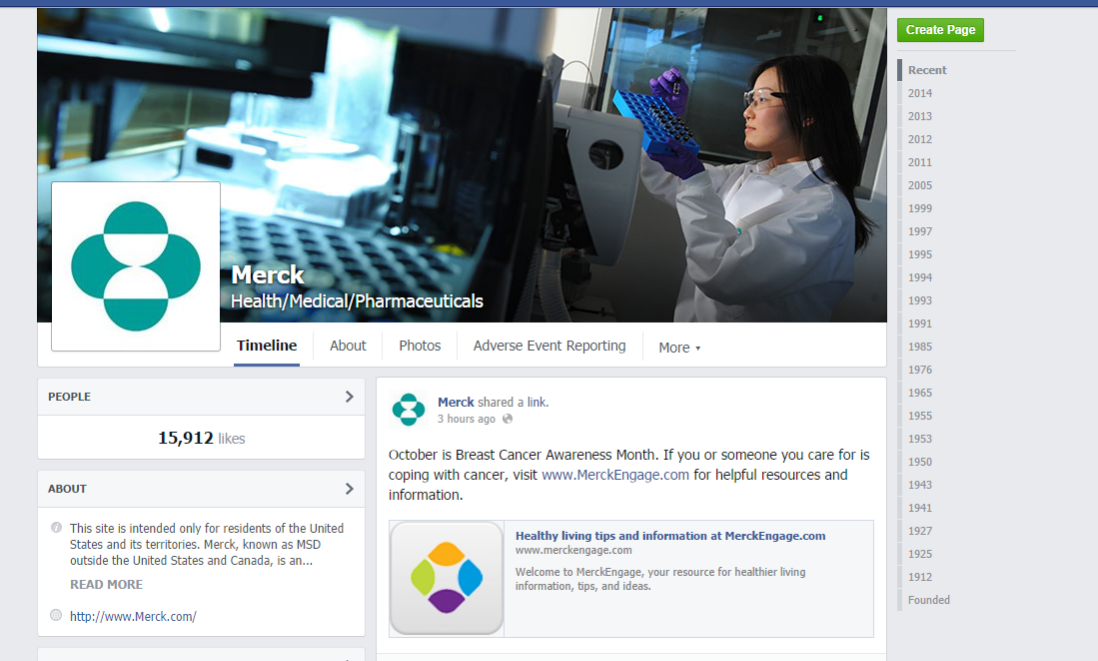
Example Facebook page from company-specific analysis.

**Figure 2:**
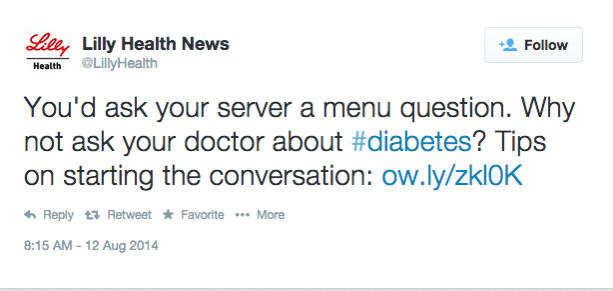
Example tweet from company-specific analysis.

We analyzed (1) the social media information on the company’s website; (2) each company’s Facebook, Twitter, and YouTube page-level characteristics (eg, overall number of followers, commenting policies); (3) randomly selected posts appearing on those pages; and (4) user-generated comments on the randomly selected posts. For the individual posts, we randomly selected 20 posts from each site during a 1-year time frame (October 1, 2013-September 30, 2014). For pages with fewer than 20 posts in the time frame, the 20 most recent posts were selected. A total of 740 posts and 348 user-generated comments were analyzed. See Figures 1 and 2 for examples of content analyzed in the company-specific analysis.

#### Drug-Specific Analysis

**Figure 3:**
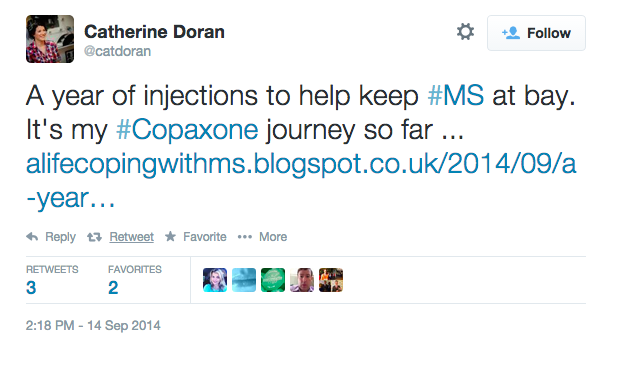
Example tweet from drug-specific analysis.

For Facebook and YouTube, each drug’s name was entered into the site’s search bar. Because most people do not venture past the first page of search results [19], the top 10 results were selected. Additionally, we collected the 10 most recent user-generated comments on the selected pages. For Twitter, we searched for each drug using a hashtag with the drug name (eg, #abilify) and randomly selected 20 tweets made within a 1-year time period (October 1, 2013-September 30, 2014). See Figure 3 for an example tweet from the drug-specific analysis. As in previous social media analyses, the sample was limited to content written in English [20,21]. A total of 800 pages/tweets/videos were analyzed.

### Company-Specific Variables

#### Social Media Sites

Any social media site links on the company’s official webpage, including Facebook, Twitter, YouTube, Google+, LinkedIn, Flickr, Instagram, Pinterest, or blogs, were recorded.

#### Audience Reach

As in other content analyses of social media, the page likes (Facebook), followers (Twitter), and subscribers (YouTube) were coded to assess audience reach [20,22].

#### Page Commenting Policy

For each page, the presence or absence of a policy for user-generated comments was recorded. If a commenting policy existed, we assessed whether it prohibited discussions of (1) drug products, (2) drug benefits, and (3) drug risks, and whether the policy stated (4) the company would remove misinformation and (5) how users should report adverse events to the FDA.

#### Post/Comment Source

We assessed whether the content was originally authored by (1) the pharmaceutical company, (2) other for-profit company, (3) media outlet (news, television, radio, etc), (4) government agency, (5) nonprofit or academic organization, (6) consumer, or (7) other source. These categories were adapted from previous social media content analyses [23,24].

#### Post/Comment Content

We coded the presence or absence of the following content for each post/comment. Using the FDA’s DTCA definitions, a post/comment could include (1) drug product claims or information about a specific drug and condition(s) it treats, (2) reminder information or information about a specific drug without uses, or (3) help-seeking information or information about a health condition without mentioning a treatment. For drug product claims, it was also noted whether the content included benefit and/or risk information. Additionally, content could include (4) nondrug treatment or information about nonpharmaceutical options to treat conditions and/or improve physical or mental health, (5) company information or news, or (6) job information/career opportunities.

#### Post/Comment Format

Based on previously used categories [19], we assessed the format of the information posted online. Information could be presented as either one or a combination of the following: (1) video, (2) image, (3) audio, and/or (4) text. Additionally, a post/comment could be an (5) interactive click-and-choose activity (poll, quiz, contest, or game) or (6) personalized/tailored content, where users receive a unique response based on provided information. We also coded whether a post/comment was presented as a testimonial (personal experience or story) or as didactic information (facts, reasons, or opinions without personal experience).

#### Post Interactivity

The interactivity of the post was assessed in multiple ways. First, following previous social media studies, we coded audience engagement as the number of “likes” (Facebook, YouTube), views (YouTube), shares (Facebook), and retweets and favorites (Twitter) [20,24]. Second, we assessed whether commenting was allowed and, if so, if the post solicited comments (ie, directly asked users to comment, retweet, or share the content) [19]. Third, the number of user-generated comments on each post and the number of company replies were recorded.

#### Comment Valence and Relevance

The valence of user-generated comments was coded as either (1) positive (ie, expressing support for the company, its products, or the content of the initial post), (2) negative (ie, expressing opposition to the company, its products, or the content of the initial post), or (3) mixed/neutral (ie, expressing both support and opposition). User-generated comments could also either be (1) relevant to the original post and on-topic or (2) irrelevant to the original post and clearly off-topic.

### Drug-Specific Variables

#### Source/Site Proprietor

In addition to using the source options from the company-specific analysis, we also noted whether the site proprietor or account holder/creator was (1) an individual, (2) pharmaceutical company/representative, (3) another organization/group, or (4) other.

#### Content

**Figure 4:**
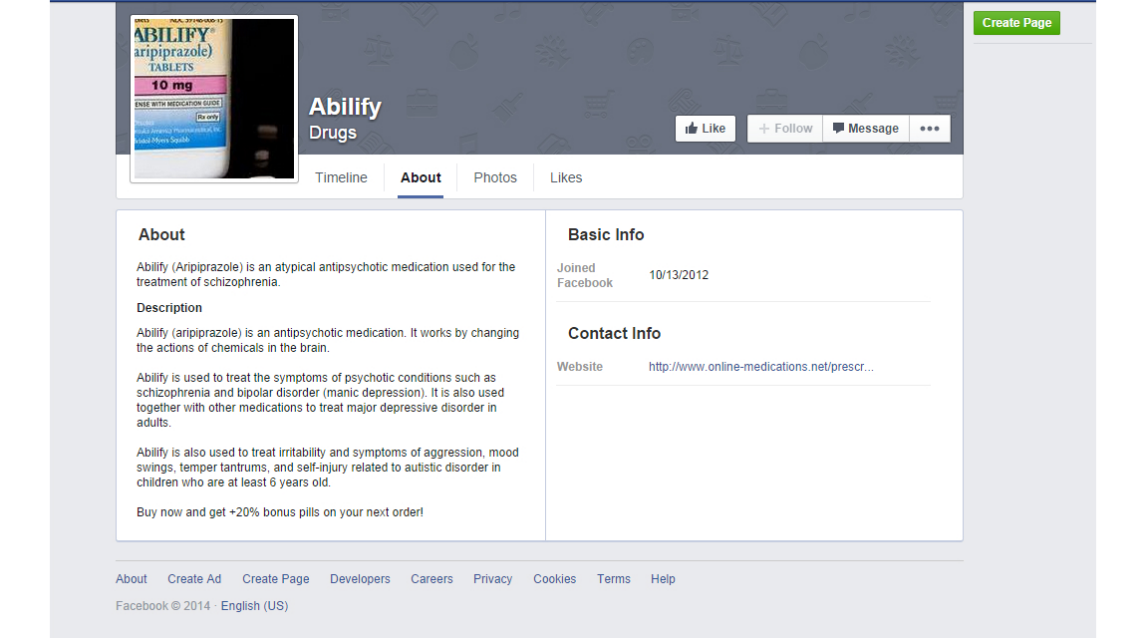
Example Facebook page from drug-specific analysis.

The presence or absence of the following information was recorded for both the main posts and the user-generated comments. First, it was assessed whether the content was actually about the drug. Additionally, the content could make a claim about the drug’s efficacy; if coded, we assessed whether the claim included benefit and/or risk information. Other content included (1) alternative treatment options, including other drugs or behaviors; (2) pharmaceutical company news; (3) emotional/informational support from other patients; (4) illegal pharmacies; and (5) lawsuits against the pharmaceutical company. See Figure 4 for an example of an illegal pharmacy on Facebook.

#### Format and Tone

The format codes from the company-specific analysis were used to classify the format of the content in the drug-specific analysis. We also coded whether the content was presented as humorous, such as joking about the side effects of the drug, or serious/nonhumorous.

### Coder Training and Intercoder Reliability

Two separate teams of 3 coders each practiced extensively to clarify definitions and coding decisions. Each coder averaged a training time of approximately 30 hours. Each team coded 10% of their respective samples for reliability testing and intercoder reliability was established for all reported variables (Krippendorff’s α>.70). For the company-specific analysis, Krippendorff’s alpha scores ranged from .73 to 1.00. For the drug-specific analysis, scores ranged from .81 to 1.00.

## Results

### Company-Specific Analysis

#### Pharmaceutical Companies’ Social Media Accounts

##### Overview

With the exception of Sinopharm, all pharmaceutical companies linked to at least one social media account on their website. Twitter was the most common social media site used (93%, 14/15), followed by Facebook (66%, 10/15), YouTube (66%, 10/15), and LinkedIn (60%, 9/15). Other less common social media sites included blogging platforms (26%, 4/15), Pinterest (26%, 4/15), Instagram (13%, 2/15), Flickr (13%, 2/15), and Google+ (6%, 1/15).

##### Company Facebook, Twitter, and YouTube Pages

The audience reach of 38 pages (10 Facebook, 17 Twitter, and 11 YouTube) was analyzed. The Facebook pages ranged in likes from 4716 to 642,816 (mean 105,806, SD 194,560; median 21,342.50, IQR 113,799). The Twitter pages ranged from 1521 to 98,589 followers (mean 36,723, SD 32,770). The YouTube accounts had a mean 2074 subscribers (SD 3169; median 924, IQR 1879), ranging from zero to 11,096 subscribers.

Across sites, the majority of pages did not have a formal commenting policy (63%, 24/38). Of the existing policies, most suggested misinformation would be removed (92%, 13/14), but did not explicitly prohibit consumers from making claims about their pharmaceutical products (85%, 12/14). The majority of policies did, however, provide information on how to report adverse events to the FDA (85%, 12/14).

##### Company Posts and User-Generated Comments

A total of 740 posts on pharmaceutical companies’ social media accounts (200 Facebook, 340 Twitter, and 200 YouTube) and 348 user-generated comments (225 Facebook, 69 Twitter, and 54 YouTube) were analyzed.

#### Electronic Direct-to-Consumer Advertising

##### Overview

Table 2 displays the percentage of posts and user-generated comments on company-run Facebook, Twitter, and YouTube pages that included information that matched the FDA’s definition of DTCA. Help-seeking information was the most common form of eDTCA; it was present in approximately 40% of all main posts (301/740), but was more commonly found on YouTube and Twitter than on Facebook (χ^2^_2_=14.6, *P=*.001). Drug product claims were present in only 1.6% of posts (12/740); of these, all posts mentioned the benefits of the drug (12/12) and only 33% (4/12) also mentioned its risks. Only 0.1% (1/740) of posts contained reminder information. Overall, most eDTCA found in pharmaceutical companies’ social media posts could be classified as help-seeking advertisements; specific information about drug products was rare. However, when drug product claims were made, the majority did not follow fair-balance rules.

**Table 2:** Electronic direct-to-consumer advertising (eDTCA) on company-run social media pages.^a^

Content				Facebook	Twitter	YouTube	Total
**Main posts, n**				200	340	200	740
	**eDTCA, n (%)**						
		Help-seeking		59 (29.5)^b^	149 (43.8)	93 (46.5)^c^	301 (40.7)
		**Drug product claims**		0 (0)	8 (2.4)	4 (2.0)	12 (1.6)
			Benefits only^d^	0 (0)	5 (63)	3 (75)	8 (67)
			Risks only^d^	0 (0)	0 (0)	0 (0.0)	0 (0)
			Benefits and risks^d^	0 (0)	3 (38)	1 (25)	4 (33)
		Reminder		1 (0.5)	0 (0)	0 (0)	1 (0.1)
**User-generated comments, n**				225	69	54	348
	**eDTCA, n (%)**						
		Help-seeking		15 (6.7)	12 (17)^c^	3 (6)	30 (8.6)
		**Drug product claims**		4 (1.8)	0 (0)	0 (0)	4 (1.1)
			Benefits only^e^	2 (50)	0 (0)	0 (0)	2 (50)
			Risks only^e^	2 (50)	0 (0)	0 (0)	2 (50)
			Benefits and risks^e^	0 (0)	0 (0)	0 (0)	0 (0)
		Reminder		2 (0.9)	0 (0)	0 (0)	2 (0.6)

^a^ Percentages in table based on column N, except where noted.

^b^ Statistically underrepresented in sample.

^c^ Statistically overrepresented in sample.

^d^ Percentages based on drug product claim posts only (n=12).

^e^ Percentages based on drug product claim comments only (n=4).

The user-generated comments on pharmaceutical companies’ social media posts followed a similar pattern. Information that matched the FDA’s definition of a help-seeking advertisement was the most common in comments (8.6%, 30/348) and was primarily found in user replies to company tweets (χ^2^_2_=8.5, *P=*.02). Drug product claim information was present in 1.1% (4/348) of comments, with half of these comments mentioning only benefits and half mentioning only risks. Similar to posts, reminder information was rare in comments (0.6%, 2/348). Overall, user-generated comments did not contain much DTCA-related information, but of those containing drug product claims, half did not provide any risk information.

##### Other Content

Table 3 displays the percentage of non-DTCA content in posts and user-generated comments on company-run Facebook, Twitter, and YouTube pages. The majority of pharmaceutical companies’ posts shared company news (63.4%, 469/740), with this information most commonly shared on Twitter (χ^2^_2_=15.3, *P<*.001). A small portion of posts shared job information (5.0%, 37/740) and approximately 15% (112/740) of posts shared nondrug treatments for improving health. User-generated comments followed a similar pattern, as company news was the most common type of content (21.3%, 74/348) followed by nondrug treatments (2.9%, 10/348) and job information (1.4%, 5/348). Overall, both pharmaceutical companies’ social media posts and user-generated comments primarily discussed company news.

**Table 3:** Non–electronic direct-to-consumer advertising (eDTCA) content on company-run social media pages.^a^

Content			Facebook	Twitter	YouTube	Total
**Main posts, n**			200	340	200	740
	**Non-eDTCA, n (%)**					
		Nondrug treatment	34 (17.0)	44 (12.9)	34 (17.0)	112 (15.1)
		Company news	138 (69.0)	190 (55.9)^b^	141 (70.5)^c^	469 (63.4)
		Job information	16 (8.0)	14 (4.1)	7 (3.5)	37 (5.0)
**User-generated comments, n**			225	69	54	348
	**Non-eDTCA, n (%)**					
		Nondrug treatment	4 (1.8)	3 (4)	3 (6)	10 (2.9)
		Company news	44 (19.6)	17 (25)	13 (24)	74 (21.3)
		Job information	5 (2.2)	0 (0)	0 (0)	5 (1.4)

^a^ Percentages in table based on column n.

^b^ Statistically underrepresented in sample.

^c^ Statistically overrepresented in sample.

##### Source

Pharmaceutical companies authored the vast majority of content on their social media sites (91.9%, 680/740). However, pharmaceutical companies also shared information from media sources (3.8%, 28/740), advocacy groups (1.8%, 13/740), government agencies (1.2%, 9/740), and other companies and groups (1.3%, 10/740). Consumers posted the majority of user-generated comments (79.6%, 277/348), although pharmaceutical company employees or representatives posted 11.8% (41/348) of the comments. Other sources of comments included advocacy groups (4.0%, 12/348) and other companies or groups (4.5%, 16/348).

##### Format and Interactivity

The majority of pharmaceutical companies’ social media posts were text-based (51.1%, 373/740) or video-based (26.3%, 199/740), and 20.0% (148/740) included both text and images. Interactive click-and-choose activities (0.1%, 1/740) and personalized/tailored content (0.1%, 1/740) were uncommon. Testimonials were used in 16.7% of posts (123/740).

Table 4 displays the degree of interaction found on the pharmaceutical companies’ social media posts. In terms of audience engagement, Facebook posts averaged 65.53 likes (SD 75.98) and 8.5 shares (SD 15.18). Tweets averaged 2.11 favorites (SD 2.94) and 3.94 retweets (SD 4.98). YouTube videos averaged 1597.38 views (SD 31,886.88) and 211.76 likes (SD 2361.65). Close to 25% of posts had comments present (177/740), with an average of 0.50 comments per post (SD 1.32). Replies from the company were less common (mean 0.03, SD 0.20). Most interaction occurred on Facebook; of the posts with comments, half were on Facebook (χ^2^_2_=74.0, *P<*.001). Additionally, Facebook posts were more likely to solicit user-generated comments (χ^2^_2_=26.5, *P<*.001) and have replies from the company (χ^2^_2_=13.8, *P=*.001). Only YouTube allowed companies to disable comments on their posts and almost half of the YouTube videos sampled (96/200) had disabled the commenting function. Overall, audience engagement with pharmaceutical companies’ social media posts was high, as users often interacted through liking and sharing the content. Additionally, a quarter of the posts included interaction through comments and pharmaceutical companies used Facebook to both solicit comments and have discussions with consumers.

**Table 4:** Interactivity on company-run social media pages.^a^

Interactivity	Facebook, n (%) n=200	Twitter, n (%) n=340	YouTube, n (%) n=200	Total, n (%) n=740
Comments allowed	200 (100.0)	200 (100.0)	104 (52.0)	644 (87.0)
Comments present	92 (46.0)^b^	57 (16.8)^c^	28 (14.0)^c^	177 (23.9)
Comments solicited	24 (12.0)^b^	9 (2.6)^c^	5 (2.5)^c^	38 (5.0)
Company replied	13 (6.5)^b^	3 (0.9)^c^	6 (3.0)	22 (3.0)

^a^ Percentages in table based on column n.

^b^ Statistically overrepresented in sample.

^c^ Statistically underrepresented in sample.

##### User-Generated Comment Format, Valence, and Relevance

Approximately 14% (47/348) of user-generated comments on pharmaceutical companies’ social media posts were testimonials. More than half of the user-generated comments were positive (186/348), 37.4% (130/348) were classified as mixed/neutral, and 9.2% (32/348) as negative. Positive comments were overrepresented on YouTube and negative comments were overrepresented on Twitter (χ^2^_2_=17.0, *P=*.002). The majority of comments were also relevant to the initial post (83.0%, 289/740). Relevant comments were overrepresented on YouTube (χ^2^_2_=8.0, *P=*.02). Positive comments were more likely to be relevant to the initial post (177/289), whereas mixed/neutral comments were more likely to be irrelevant (42/59; χ^2^_2_=42.6, *P<*.001). The majority of positive comments were on pages with a commenting policy (153/186), whereas most negative comments were on pages without a commenting policy (19/32; χ^2^_2_=8.7, *P=*.01). There was no relationship between comment relevance and presence of a commenting policy (χ^2^_1_=0.1, *P=*.75). Overall, it appeared that user-generated comments were mostly supportive of the pharmaceutical company and its products, particularly when the company had a commenting policy in place.

### Drug-Specific Analysis

Of the 800 Facebook pages, tweets, and YouTube videos sampled from social media searches for pharmaceutical drugs, 86.9% (695/800) were actually about the searched-for drug. The following analyses included this portion of the sample.

#### Source/Site Proprietor

Of the 695 main posts about the searched-for drug, the majority of site proprietors were individuals (51.1%, 355/695) or nonpharmaceutical organizations (48.3%, 336/695). Pharmaceutical companies ran 0.6% of accounts (4/695). On Twitter and YouTube, consumers created most of the content (41.1%, 248/603), closely followed by media sources (37.0%, 233/603). Other sources included advocacy groups (6.0%, 36/603), pharmaceutical companies (3.3%, 20/603), other for-profit companies (9.0%, 54/603), and government agencies (1.0%, 6/603). Overall, most information from searches for drugs on Facebook, Twitter, or YouTube was attributed to members of the public rather than pharmaceutical companies.

#### Drug Product Claims

Table 5 displays the percentage of drug product claims in the search results on Facebook, Twitter, and YouTube. The majority included drug product claims (69.4%, 482/695), most of which were on YouTube (χ^2^_2_=13.7, *P=*.001). Of the drug product claims, posts mentioning only the benefits (44.8%, 216/482) were significantly more common than both risk-only posts (27.2%, 131/482) and posts that discussed both benefits and risks (28.0%, 135/482; χ^2^_2_=28.6, *P<*.001). The majority of user-generated comments on Facebook and YouTube videos also contained drug product claims (85.4%, 140/164). In contrast to the main posts, risk-only information (39.2%, 55/140) was significantly more common in comments than benefit-only information (22.9%, 32/140; χ^2^_2_=7.0, *P=*.03). Overall, results indicate that when the public searches for drugs on Facebook, Twitter, or YouTube, they are likely to come into contact with claims about those drugs’ effectiveness. Although the main posts often highlight the benefits of the drug, the user-generated comments often present a contrasting view.

**Table 5:** Drug product claims in the drug-specific analysis.^a^

Content			Facebook	Twitter	YouTube	Total
**Main posts, n**			92	409	194	695
	**Drug product claims, n (%)**		68 (73.9)	262 (64.1)	152 (78.4)^b^	482 (69.4)
		Benefits only^c^	24 (35.5)	148 (56.5)^b^	44 (28.9)^d^	216 (44.8)
		Risks only ^c^	14 (20.6)^b^	85 (32.4)^b^	32 (21.1)^d^	131 (27.2)
		Benefits and risks^c^	30 (44.1)^b^	29 (11.1)^d^	76 (50.0)^b^	135 (28.0)
**User-generated comments, n**			51		113	164
	**Drug product claims, n (%)**		46 (90)		94 (83.1)	140 (85.4)
		Benefits only^e^	15 (33)		17 (18.1)	32 (22.9)
		Risks only^e^	14 (30)		41 (43.6)	55 (39.3)
		Benefits and risks^e^	17 (37)		36 (38.3)	53 (37.8)

^a^ Percentages in table based on column n, except where noted.

^b^ Statistically overrepresented in sample.

^c^ Percentages in row based on drug product claim posts only (n=482).

^d^ Statistically underrepresented in sample.

^e^ Percentages in row based on drug product claim comments only (n=140).

#### Illegal Pharmacies

Table 6 presents the other content found in the social media search results. Illegal pharmacies were present in 17.4% (16/92) of Facebook pages. Illegal pharmacies were less common on YouTube (χ^2^_2_=29.6, *P<*.001). Links to illegal pharmacies were also present in 9.1% (15/164) of user-generated comments on Facebook and YouTube; these comments were also more common on Facebook (χ^2^_1_=13.7, *P<*.001). When searching for drug information on social media, consumers were likely to come into contact with at least one link to an illegal pharmacy, particularly if consumers conducted the search on Facebook.

**Table 6:** Other content in drug-specific analysis.^a^

Content		Facebook	Twitter	YouTube	Total
**Main posts, n**		92	409	194	695
	Illegal pharmacies, n (%)	16 (17)^b^	21 (5.1)	3 (1.5)^c^	40 (5.8)
	Lawsuits, n (%)	11 (12)^b^	26 (6.4)	2 (1.0)^c^	39 (5.6)
	Patient support, n (%)	8 (9)	6 (1.5)^c^	32 (16.5)^b^	46 (6.6)
	Alternative treatments, n (%)	6 (7)	16 (3.9)	8 (4.1)	30 (4.3)
	Company news, n (%)	20 (22)	100 (24.4)^b^	11 (5.7)^c^	131 (18.8)
**User-generated comments, n**		51		113	164
	Illegal pharmacies, n (%)	11 (22)^b^		4 (3.5)^c^	15 (9.1)
	Lawsuits, n (%)	10 (20)^b^		1 (0.9)^c^	11 (6.7)
	Patient support, n (%)	12 (24)^c^		47 (41.6)^b^	59 (36.0)
	Alternative treatments, n (%)	7 (14)		27 (23.9)	34 (20.7)
	Company news, n (%)	13 (26)^b^		3 (2.7)^c^	16 (9.8)

^a^ Percentages in table based on column n.

^b^ Statistically overrepresented in sample.

^c^ Statistically underrepresented in sample.

#### Other Content

Lawsuit information was present in 5.6% (39/695) of all drug-specific social media posts and was more common on Facebook and Twitter than YouTube (χ^2^_2_=15.1, *P=*.001). Patient support (6.6%, 46/695) and alternative treatment information (4.3%, 30/695) were present in fewer posts than company news (18.8%, 131/695). The majority of patient support was on YouTube (χ^2^_2_=48.8, *P<*.001). Most of the company news was on Twitter (χ^2^_2_=30.9, *P<*.001). There was no difference in alternative treatment information based on social media site (χ^2^_2_=1.3, *P=*.53). Thus, when searching for drug information on social media sites, consumers were likely to find information about the pharmaceutical company on Twitter, but support from other patients on YouTube. Information regarding lawsuits was found slightly less often than illegal pharmacies on Facebook. Alternative treatment options were relatively uncommon on all social media sites.

In contrast to the posts, patient support (36.0%, 59/164) and alternative treatment information (20.7%, 34/164) were more common than company news (9.8%, 16/164) and lawsuit information (6.7%, 11/164) in user-generated comments on Facebook and YouTube. Lawsuits (χ^2^_1_=19.7, *P*<.001) and company news (χ^2^_1_=20.8, *P<*.001) were more common on Facebook, whereas support was more common on YouTube (χ^2^_1_=5.0, *P=*.04). Overall, these results indicate that other consumers commented to provide alternative treatment options and support, even though this content was largely absent in the main posts.

#### Reach, Format, and Tone

Approximately 25% of posts on Twitter and YouTube were testimonials (150/603). Additionally, a large majority of tweets and YouTube videos had a serious, nonhumorous tone (96.7%, 583/603). The Facebook pages ranged from zero to 62,427 likes (median 69.0, IQR 328.3). Approximately 80% of tweets had zero favorites and zero retweets, with a mean of 0.42 favorites (SD 1.53) and 1.25 retweets (SD 16.32). YouTube video views ranged from 2 to 1,077,399 (median 4707.0, IQR 14,009). YouTube video likes ranged from zero to 1671 (median 51.83, IQR 30.0). In general, the majority of posts that arose from searches about specific drugs on social media provided didactic, nonhumorous information. The degree of audience engagement with drug information on social media sites varied widely.

## Discussion

### Principal Findings

The results of this study directly address critical concerns raised by researchers and public health officials about the marketing of pharmaceutical drugs via social media. Importantly, novel evaluations are provided about (1) how pharmaceutical companies use social media for DTCA, (2) how greatly companies reach and interact with consumers through social media, and (3) how likely people are to be exposed to drug efficacy claims and information about illegal pharmacies when searching for information about pharmaceutical drugs via social media. Respectively, the results suggest that (1) pharmaceutical companies avoid making drug product claims but frequently post help-seeking content, (2) thousands of people often view and share content posted by pharmaceutical companies, and (3) people are likely to be exposed to drug product claims and information about illegal pharmacies when searching for information about popular pharmaceutical drugs on social media.

More specifically, approximately 40% of all pharmaceutical companies’ Facebook, Twitter, and YouTube posts in our sample met the FDA’s definition of a help-seeking advertisement. This content focuses on generating awareness of a health condition or disease and often suggests that the audience should learn about potential treatment options from their doctor or other source. Despite concerns that specific drugs would be heavily advertised through pharmaceutical companies’ social media accounts, product claim advertisements were uncommon. Only approximately 1% of posts contained a product claim. However, one-third of the product claim posts did not include any information on drug risks, thus failing to adhere to FDA regulations for traditional DTCA. Although this occurred in a relatively small number of posts overall, the problems surrounding the absence of risk information in product claims is well documented [2,4,5,25]. The FDA has developed draft guidelines for eDTCA regulations [26], and the inclusion of risk information is required for all company postings about specific products. To increase compliance with fair-balance rules on social media, the FDA should finalize the eDTCA regulations and formally detail how regulatory oversight will be enacted. Although monitoring every single post is likely unfeasible, the FDA could follow the procedures of this study to regularly monitor a random selection of posts and require pharmaceutical companies to notify the FDA whenever they use any media to share information with the public that is consistent with traditional forms of DTCA.

It is particularly important for the FDA to monitor pharmaceutical companies’ social media accounts because they can have rather large audiences. Pharmaceutical companies’ social media pages averaged approximately 45,000 followers or subscribers. Additionally, our results indicate that audience members are actively interacting with companies and sharing the content that the companies’ post with people in their own social networks. For instance, posts are often liked and shared on Facebook and favorited and retweeted on Twitter. The public approval of this information on users’ social network pages increases the potential for these posts to influence a large portion of the public. For example, research suggests that health behaviors and attitudes often spread through social networks and a number of social media-based interventions have shown that exposure to health information on social networking sites leads to health behavior change [27,28]. Thus, the messages that pharmaceutical companies share through social media channels have the potential to reach and influence millions of people worldwide; estimates of direct exposure grossly underestimate the cumulative influence of eDTCA on social media.

One common concern regarding eDTCA is that positive (and potentially misleading) product claim testimonials would populate the user-generated comments on pharmaceutical companies’ pages [3,4]. Approximately 25% of posts had at least one comment and most were supportive of the company. However, very few comments contained information that would be classified as DTCA if the pharmaceutical company had produced the comments. Most commonly, user-generated comments contained information that resembled content that would appear in a help-seeking ad. When users did make drug product claims, however, they tended to focus either exclusively on benefits or risks. Additionally, most companies did not explicitly prohibit users from making product claims in their commenting policies. According to current FDA draft guidance documents, pharmaceutical companies are not responsible for the content of user-generated comments unless the comments were created by, paid for, or edited by the company [29,30]. Most commenting policies did, however, suggest that inaccurate information would be removed. Under current FDA draft guidance documents, pharmaceutical companies can, but are not required to, correct misinformation about their products in user-generated comments [30]. Interestingly, companies with commenting guidelines had significantly more positive comments than those without a commenting policy. Although it cannot be determined through this analysis, companies who are more aware of user-generated comments (and thus have a commenting policy) might be deleting negative user-generated comments [4]. If companies selectively delete user-generated comments, the information in the remaining user-generated comments would be applicable to FDA regulations [29,30].

Although uncommon on pharmaceutical companies’ sites, product claims and testimonials were largely present in posts resulting from general searches for drug information on Facebook, Twitter, and YouTube. The majority of the top search results contained drug efficacy claims. Troublingly, most claims were made by nonexpert sources and mentioned only benefits of the drug rather than presenting a balanced view of both the benefits and risks of product use. Given that approximately 20% of Internet users check online reviews of particular drugs [31], our results suggest that consumers are likely getting incomplete drug information through social media. Additionally, around 25% of these posts were testimonials, a format that often enhances the credibility of the claims made [3-5]. Furthermore, it is likely that well-known and trusted media such as Facebook lend credibility to the health information posted within their pages relative to other online channels [9]. For example, young adults, the most prolific users of social networking sites [6], are the most likely age group to trust health information on social media [32] and to search for health advice and others’ health experiences on social media [13].

The potential credibility afforded to information on social media is also problematic when we consider the continued presence of illegal pharmacies in the top search results. Illegal pharmacies were most prominent on Facebook; approximately 20% of the Facebook pages in the drug-specific analysis advertised illegal pharmacies. Although Facebook’s terms of use prohibit illegal activity [33] and the organization is partnered with the Center for Safe Internet Pharmacies [8], our results suggest Facebook is not adequately policing its site for illegal pharmacies. We echo the calls of other scholars for social media companies to actively monitor their sites and make meaningful policy changes to eliminate this type of content [7,8,15]. Past policy changes demonstrate that social media organizations can have a measurable impact on the presence of pharmaceutical drug information on their sites. For example, Facebook eliminated companies’ ability to block the commenting feature on their pages in 2011, so many of the drug product pages that existed in previous analyses [12] were discontinued [3]. Thus, these sites need to take an active role in protecting their users from harmful illegal pharmacies.

### Limitations

There are some limitations to the present study. First, we focused our analysis on the top pharmaceutical companies and best-selling drugs. As a cost-effective marketing strategy, smaller companies might rely on social media advertising more so than larger companies and pharmaceutical companies might use social media channels to introduce newer, less established drugs to the marketplace [3]. Additionally, although we analyzed a large number of postings, our review only focused on 1 year of social media activity. As marketing trends constantly change [12], future research should investigate if the presence of eDTCA changes over time. Last, we focused on the presence of product claims, benefits, and risks and did not examine the accuracy of the claims or whether benefits or risks were emphasized within in a single post. To get a more complete picture of the pharmaceutical drug information that appears on social media sites, future research should explore these areas.

### Conclusions

Social media sites are an accessible channel for pharmaceutical companies and others to easily deliver drug information to millions of people across the globe. Although pharmaceutical companies are not directly marketing specific products through their social media accounts often, they are posting content similar to help-seeking DTCA, which describes a health condition without providing a specific solution. If people search for drug solutions to these ailments via social media sites, they will likely be exposed to testimonials that highlight pharmaceutical drug benefits over risks as well as links to pharmacies where they can illegally purchase these drugs. Thus, pharmaceutical drug information on social media sites is potentially quite dangerous to public health and should be monitored accordingly.

## Abbreviations

DTCA: direct-to-consumer advertising
eDTCA: electronic direct-to-consumer advertising
FDA: Food and Drug Administration
